# Voltage-Mode Multifunction Biquad Filter and Its Application as Fully-Uncoupled Quadrature Oscillator Based on Current-Feedback Operational Amplifiers

**DOI:** 10.3390/s20226681

**Published:** 2020-11-22

**Authors:** San-Fu Wang, Hua-Pin Chen, Yitsen Ku, Ming-Xiu Zhong

**Affiliations:** 1 Department of Electronic Engineering, National Chin-Yi University of Technology, Taiping, Taichung 41170, Taiwan; sf_wang@ncut.edu.tw; 2 Department of Electronic Engineering, Ming Chi University of Technology, New Taipei 24301, Taiwan; M08158016@o365.mcut.edu.tw; 3 Department of Electrical Engineering, California State University Fullerton, Fullerton, CA 92831, USA; joshuaku@fullerton.edu

**Keywords:** active filter, current-feedback operational amplifiers (CFOAs), oscillators, circuit design

## Abstract

This research introduces a new multifunction biquad filter based on voltage mode (VM) current-feedback operational amplifier (CFOA) and a fully uncoupled quadrature oscillator (QO) based on the proposed VM multifunction biquad filter. The proposed VM multifunction biquad filter has high impedance to the input voltage signal, and uses three CFOAs as active components, while using four resistors and two grounded capacitors as passive components. The VM CFOA-based multifunction biquad filter realizes band-reject, band-pass, and low-pass transfer functions at high-input impedance node simultaneously, which has the feature of easy cascading in VM operation without the need for additional voltage buffers. Additionally, the filter control factor parameter pole frequency (ω_o_) and quality factor (Q) of the proposed VM multifunction biquad filter can be independently set by varying different resistors. By slightly modifying the VM multifunction biquad filter topology, a VM fully-uncoupled QO is easily obtained. The difference from the previous VM CFOA-based multifunction biquad filter is that the proposed VM CFOA-based multifunction biquad filter can be independently controlled by the filter control factor parameters, ω_o_ and Q. The proposed VM CFOA-based multifunction biquad filter can be transformed into a VM QO with fully-uncoupled adjustable of the oscillation condition and the oscillation frequency. The oscillation condition and the oscillation frequency can be fully-uncoupled and controlled by varying two sets of completely different resistors. The proposed VM fully-uncoupled QO solves the amplitude instability. The constant amplitude ratio of two quadrature sinusoidal waveforms can be realized when tuning FO. PSpice simulation and experimental results prove the performances of the proposed VM multifunction filter and VM fully-uncoupled QO. Simulation and experimental results confirm the theoretical analysis of the proposed circuits.

## 1. Introduction

**Figure 1:**
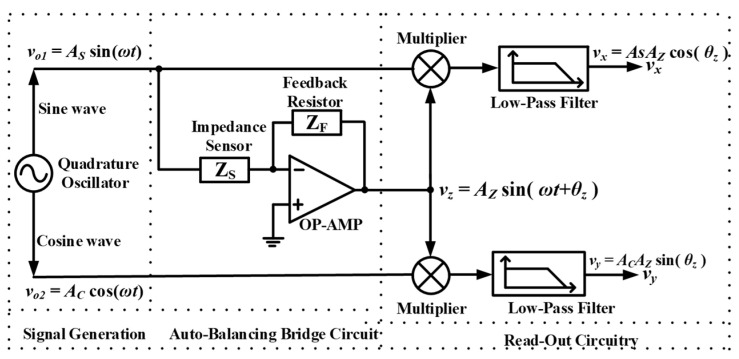
The conceptual scheme of a dual PSD system.

Voltage-mode (VM) analog active filters and oscillators using different active components have received extensive attention. Some innovative approaches to realize VM biquad filters [1,2,3,4] and oscillators [5,6,7,8,9] can be found in the open study. Single-input three-output VM multifunction biquad filters, such as band-pass filter (BPF), low-pass filter (LPF), high-pass filter (HPF) or band-reject filter (BRF), are applied to the phase-locked loop, the high fidelity 3-way speaker, the touch-tone telephone tone decoder, and the phase sensitive detection (PSD) [8,9,10,11]. PSD is suitable for detecting and measuring low frequency electrical signal from the sensor [8,9]. The conceptual scheme of a dual PSD including a VM quadrature oscillator (QO), an auto-balancing bridge circuit, two multiplier circuits and two LPF circuits module is shown in Figure 1 [8,9,10]. VM QO can generate two sinusoidal wave output with a 90° phase difference and is the great significant part of a PSD [8,9]. Based on the dual PSD system, low-frequency QOs and filters research are required. The potential applications and advantages of using current-feedback operational amplifiers (CFOAs) to design VM biquads have attracted considerable attention in [12,13,14,15,16,17,18,19,20,21,22,23,24,25,26,27]. CFOA can be obtained by the cascade of a positive second-generation current conveyor and a voltage follower, so it can be implemented by the commercially integrated circuit (IC) namely Analog Device AD844AN [28,29]. The voltage on the non-inverting input port +IN of AD844AN is transferred to the inverting input port -IN, and the current flowing to the inverting port -IN is replicated to the port Tz. The voltage output port O follows the port Tz voltage. Compared with conventional operational amplifier (OP-AMP), AD844AN has the advantages of wider bandwidth and higher slew rate [28,29]. Therefore, for a specific purpose design, a dual PSD system, or a small number of circuits, using a commercially available ICs to design a VM biquad filters and QOs is better choice. According to the AD844AN datasheet [29], the rated temperature range of AD844AN is −40 °C to +85 °C, which is industrial temperature range, and the power supply range of AD844AN is ±4.5 V to ±18 V. Hence, when the circuits operate at different temperatures, the performances of the circuits will not have obvious fluctuations. Based on the above advantages, AD844AN has been widely used in the open literature [12,13,14,15,16,17,18,19,20,21,22,23,24,25,26,27,28,29,30,31,32].

As mentioned above, it is beneficial to use CFOA as an active component to implement a variety of high-input impedance VM multifunction biquads. The VM biquads, which has a high impedance for the input voltage signal, has aroused great interest because this biquads can be easily cascaded without any voltage buffer [21,22,23,24,25,26,27]. Singh and Senani [23] proposed a VM multifunction biquad HPF, LPF, and BPF transfer functions, but it employed four CFOAs and eight passive components. Horng and Lee [24] proposed a VM multifunction biquad HPF, LPF, and BPF transfer functions using three CFOAs and seven passive components, but it had the disadvantage of using three capacitors. Shan and Malik [25] proposed another VM multifunction biquad BRF, BPF, LPF, and HPF transfer functions using four CFOAs and six passive components, but it had the disadvantage of using four CFOAs. CFOA-based VM multifunction biquad HPF, LPF, and BPF has been proposed [26]. The transfer functions of the proposed filter use three CFOAs and four passive components, where one of the X terminals of the CFOA is connected to a grounded capacitor, which will lead to an improper transfer function and poor performance at high frequency. In 2019, a CFOA-based high-input impedance VM multifunction biquad has been proposed [27]. This circuit has important advantages, such as using only three CFOAs and realizing the transfer functions of BRF, BPF and LPF at the same time. In addition, the circuit also has the advantages of single input and three outputs, high-input impedance, and quadrature adjustable pole frequency (ω_o_) and quality factor (Q), and easily converted to VM QO. However, a further advantage cannot be achieved in [27], that is, the independent tunability of the filter control factor parameters ω_o_ and Q. Although the topology of VM multifunction biquad [27] can be converted to VM QO, it cannot achieve a fully-uncoupled tuning methods, and cannot obtain the condition of oscillation (CO) and the frequency of oscillation (FO). Note that only when CO and FO are determined by two completely different sets of components, CO and FO are called completely decoupled [30].

This research proposes a new topology for the realization of an independently tunable VM multifunction biquad filter. The proposed topology uses three CFOAs as active components, while using four resistors and two grounded capacitors as passive components. The advantages of the proposed CFOA-based VM multifunction biquad filter are follows: (i) use only three CFOAs, (ii) use only two grounded capacitors, (iii) realize three standard filter transfer functions with one input and three outputs at the same time, (iv) high-input impedance, (v) the input parasitic resistances of the X ports of the CFOAs can be easily accommodated in an external resistors, (vi) independent control of the filter control factor parameters ω_o_ and Q, and (vii) transformed into a VM QO with fully-uncoupled adjustable of CO and FO. Table 1 compares the proposed VM multifunction biquad filter with previously published researches [12,13,14,15,16,17,18,19,20,21,22,23,24,25,26,27]. It can be seen that the proposed VM multifunction biquad filter can simultaneously achieve all the above imported properties. Unlike the recently reported in [27], the attractive feature of the proposed VM multifunction biquad filter can be controlled independently of the control factor parameters ω_o_ and Q, and transformed into a VM QO with fully-uncoupled adjustable of CO and FO. Furthermore, the proposed VM QO with fully-uncoupled is advantageous to achieve amplitude stability. Table 2 summarizes the performance of the filter and oscillator, and compares the specific characteristics of the study [27].

In this research, a new VM multifunction biquad filter with high-input impedance and a VM fully-uncoupled QO using the proposed VM multifunction biquad filter are presented. Compared with the previous research [27], the proposed VM multifunction biquad filter can overcome the independent control of the filter control factor parameters ω_o_ and Q, and the proposed VM CFOA-based QO can also overcome the fully-uncoupled adjustability of CO and FO. The CFOA-based biquad filter and QO are suitable for PSD system based on the use of commercially available ICs. The filter control factor parameters ω_o_ and Q are independently tuned and controlled. The QO control factor parameters CO and FO are fully-uncoupled tuning controlled. The effective frequency ranges of the bqiaud filter circuit is around 1 MHz, and the QO oscillation frequency varies from 8.16 to 628 kHz. Moreover, the proposed QO with fully-uncoupled is advantageous to achieve amplitude stabilization. The remaining sections of the research is structured as follows. Section 2 will introduce the characteristics and non-ideality of the VM CFOA-based multifunction biquadratic filter. Subsequently, based on the proposed VM CFOA-based multifunction filter, the VM fully-uncoupled QO is introduced. Section 3 verifies the proposed VM CFOA-based circuits and the theoretical comparison between experimental data and simulation data. Finally, Section 4 will summarize the research.

## 2. Proposed VM CFOA-Based Circuits 

### 2.1. Proposed VM CFOA-Based Multifunction Biquad Filter

**Figure 2:**
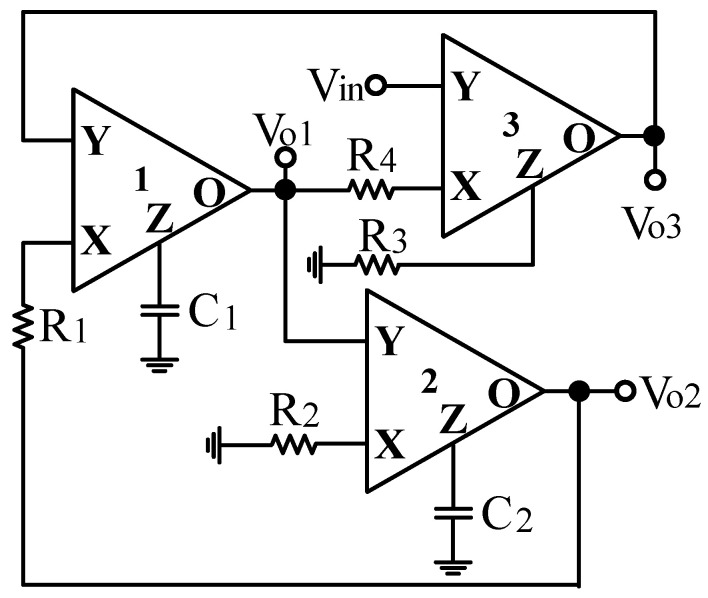
Proposed VM multifunction biquad filter.

CFOA is a four-port versatile active component and its commercially available IC is AD844-type CFOA. The four-port characteristic of CFOA can be described by V_X_ = V_Y_, V_O_ = V_Z_, I_Y_ = 0 and I_X_ = I_Z_ [31,32]. Figure 2 shows the proposed CFOA-based VM multifunction biquad filter with high-input impedance, including three CFOAs as active components, four resistors, and two grounded capacitors. Using only two grounded capacitors is particularly attractive for IC implementation. Routine analysis of the proposed filter yields the following BPF, LPF, and BRF voltage transfer functions.

(1) $$\frac{V_{o1}}{V_{\mathrm{in}}}=\frac{s(\frac{R_{3}}{C_{2}R_{1}R_{4}})}{s^{2}+s\frac{R_{3}}{C_{1}R_{1}R_{4}}+\frac{1}{C_{1}C_{2}R_{1}R_{2}}}$$

(2) $$\frac{V_{o2}}{V_{\mathrm{in}}}=\frac{\left(\frac{R_{3}}{R_{4}})(\frac{1}{C_{1}C_{2}R_{1}R_{2}}\right)}{s^{2}+s\frac{R_{3}}{C_{1}R_{1}R_{4}}+\frac{1}{C_{1}C_{2}R_{1}R_{2}}}$$

(3) $$\frac{V_{o3}}{V_{\mathrm{in}}}=\frac{\left(\frac{R_{3}}{R_{4}}{)(s}^{2}+\frac{1}{C_{1}C_{2}R_{1}R_{2}}\right)}{s^{2}+s\frac{R_{3}}{C_{1}R_{1}R_{4}}+\frac{1}{C_{1}C_{2}R_{1}R_{2}}}$$

As shown in Equations (1) to (3), the biquadratic BPF transfer function is obtained from V_o1_, the biquadratic LPF transfer function is obtained from V_o2_, and the biquadratic BRF transfer function is obtained from V_o3_. The pass-band gain of the biquadratic BPF transfer function is unity. The pass-band gains, G_LP_ and G_BR_, of biquadratic LPF and BRF transfer functions are given by

(4) $$G_{\mathrm{LP}}=G_{\mathrm{BR}}=\frac{R_{3}}{R_{4}}$$

According to the denominator polynomial of the transfer functions of the CFOA-based VM multifunction biquad filter given in Equations (1) to (3), the filter control factor parameters Q and ω_o_ of the filter can be calculated as

(5) $$Q=\frac{R_{4}}{R_{3}}\sqrt{\frac{C_{1}R_{1}}{C_{2}R_{2}}},\;\omega_{o}=\sqrt{\frac{1}{C_{1}C_{2}R_{1}R_{2}}}$$

Based on Equation (5), the following techniques for obtaining independent control of Q and ω_o_ can be suggested. By changing R_3_ and/or R_4_, the control factor parameter Q can be independently controlled without disturbing ω_o_. For fixed-value capacitors, by simultaneously changing R_1_ and R_2_ while keeping the ratio of R_1_ and R_2_ constant, the control factor parameter ω_o_ can be independently controlled without disturbing Q. Thus, the CFOA-based VM multifunction biquad filter has independent tuning capability for the filter control factor parameters ω_o_ and Q. Assuming that C_1_ = C_2_ = C and R_1_ = R_2_ = R, the filter control factor parameters of Q and ω_o_ in Equation (5) become

(6) $$\omega_{o}=\frac{1}{\mathrm{CR}},\;Q=\frac{R_{4}}{R_{3}}$$

Equation (6) describes that the control factor parameter ω_o_ can be independently controlled by changing R, and the control factor parameter Q can be independently controlled by changing R_3_ and/or R_4_. Hence, the filter control factor parameters of Q and ω_o_ of the CFOA-based VM multifunction biquad filter can be independently controlled.

**Figure 3:**
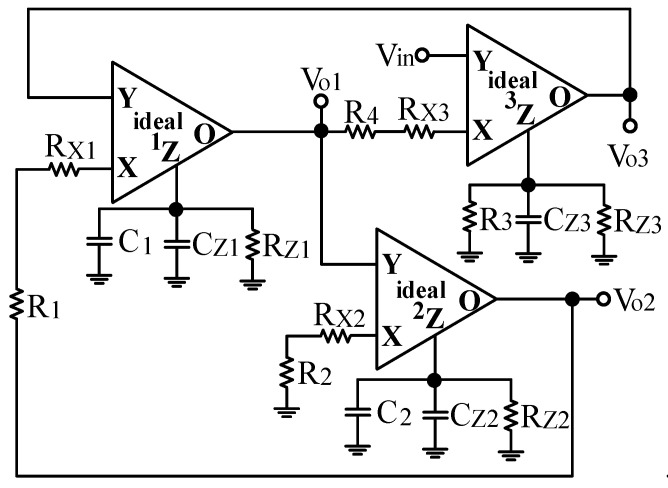
Proposed VM multifunction biquad filter including the CFOA parasitic impedances.

Next, the parasitic impedances of non-ideal CFOA is studied. The non-ideal CFOA model has parasitic resistances and capacitances from the Y port and Z port to the ground, and a series parasitic resistance R_X_ at the port X. The parasitic impedances of non-ideal CFOA are R_Yj_//C_Yj_ of port Y_j_, R_Zj_//C_Zj_ of port Z_j_, and R_xj_ of port X_j_ where j = 1, 2, 3 and represents the jth non-ideal CFOA [22]. Taking into account the parasitic impedances of non-ideal CFOA, the biquad filter presented in Figure 2 is modified to Figure 3. The proposed VM multifunction biquad filter employs external capacitors C_1_ and C_2_ connected in parallel at the first and second CFOA Z ports, respectively. This method has the characteristic that the parasitic capacitance, C_Z_, is directly incorporated into Z terminal of CFOA as a part of the main capacitance. Hence, C_1_ and C_2_ can be selected to increase the parasitic capacitances at the Z ports of CFOAs. Each X port of the CFOA is directly connected to a resistor. This method has the feature of incorporating parasitic resistance, R_X_, directly into the X port of CFOA as a part of the main resistance. However, the parasitic resistances at the Z port of CFOA will change the type of the impedances. If the following conditions can be satisfied, the influence of the non-ideal CFOA parasitic impedances in Figure 3 can be ignored.

(7) $$\frac{1}{{s(C}_{1}+C_{Z1})}<<R_{Z1}$$

(8) $$\frac{1}{{s(C}_{2}+C_{Z2})}<<R_{Z2}$$

(9) $$R_{3}<<\frac{R_{Z3}}{1+{\mathrm{sR}}_{Z3}C_{Z3}}$$

### 2.2. Proposed Fully-Uncoupled VM QO

**Figure 4:**
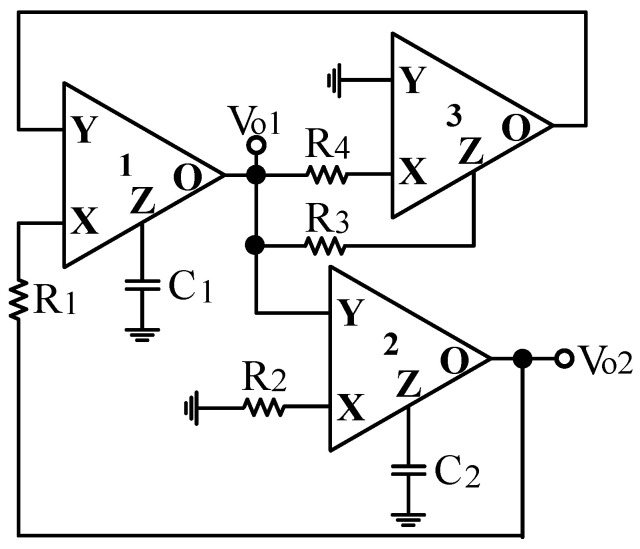
The proposed VM QO with fully-uncoupled controlled of CO and FO.

Based on the VM multifunction biquad filter structure in Figure 2, the input signal V_in_ is grounded, the grounding resistor R_3_ is floating, and the floating terminal of the resistor R_3_ is connected to the output voltage signal V_o1_. Thus, the CFOA-based VM multifunction biquad filter can be transferred to the VM fully-uncoupled QO as shown in Figure 4. Routine analysis of the proposed VM fully-uncoupled QO results in the following characteristic equation.

(10) $$s^{2}+s\frac{1}{C_{1}R_{1}}(\frac{R_{3}}{R_{4}}-1)+\frac{1}{C_{1}C_{2}R_{1}R_{2}}=0$$

According to Equation (10), the CO and FO of Figure 4 are obtained as:

(11) $$\mathrm{CO}:\;R_{3}\leq R_{4}$$

(12) $$\mathrm{FO}:\;\omega_{o}=\sqrt{\frac{1}{C_{1}C_{2}R_{1}R_{2}}}$$

Equations (11) and (12) illustrate that by controlling R_3_ and/or R_4_, CO can be tuned fully independently without affecting FO. Similarly, by controlling R_1_ and/or R_2_, FO can be tuned fully independently without affecting CO. This means that both CO and FO can be fully-uncoupled controlled by adjusting two sets of completely different resistors. The QO output voltages V_o1_ and V_o2_ are given by

(13) $$V_{o1}={\mathrm{sC}}_{2}R_{2}V_{o2}$$

In the steady state, the QO output voltages V_o1_ and V_o2_ are expressed as

(14) $$V_{o1}=\omega_{o}C_{2}R_{2}e^{j\varphi }V_{o2}$$

where the phase difference φ = 90° to ensure that the output voltages, V_o1_ and V_o2_ are in quadrature phase shifted.

From Equation (14), the magnitude ratio of the quadrature output voltages V_o1_ and V_o2_ is given by

(15) $$\left|\frac{V_{o1}}{V_{o2}}\right|=\omega_{o}C_{2}R_{2}=\sqrt{\frac{C_{2}R_{2}}{C_{1}R_{1}}}$$

Assuming that C_1_ = C_2_ and R_1_ = R_2_, the magnitude ratio of the QO output voltages V_o1_ and V_o2_ in Equation (15) becomes

(16) $${\left|\frac{V_{o1}}{V_{o2}}\right|}_{C_{1}=C_{2}{,\;R}_{1}=R_{2}}=1$$

Equation (16) describes that the phase shift of the two quadrature output voltages is 90°, and the magnitude ratio of two quadrature output voltages is also equal. Thus, the proposed VM fully-uncoupled QO solves the amplitude instability and improves the unbalance of the generated quadrature output amplitudes V_o1_ and V_o2_. When tuning FO, the constant amplitude ratio of two quadrature sinusoidal waveforms can be realized. Hence, the proposed VM fully-uncoupled QO is advantageous to the stability of the combined amplitude.

The phase noise figure-of-merit (FoM) for oscillators summarizes the important performance parameters. The conventional phase noise FoM of the oscillators is defined as follows [33].

(17) $$\mathrm{FoM}(\Delta \omega )=-L(\Delta \omega )+20\log(\frac{\omega_{o}}{\Delta \omega })-10\log(\frac{P_{\mathrm{DC}}}{1\mathrm{mW}})$$

where Δω is the offset frequency relative to the carrier, ω_o_ is the oscillation frequency, and L(Δω) is the phase noise at the offset frequency to the carrier. P_DC_ is the power (in mW) consumed by the oscillator. In order to estimate FoM of the proposed VM fully-uncoupled QO, the phase noise FoM will be discussed in the next Section.

## 3. Simulation and Experimental Results 

### 3.1. Test Setup 

**Figure 5:**
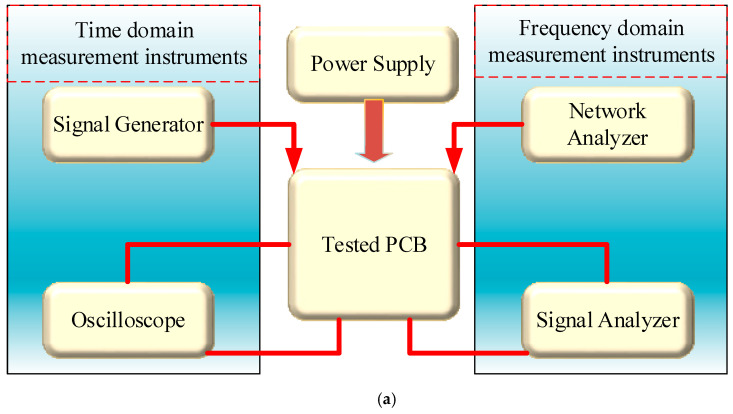

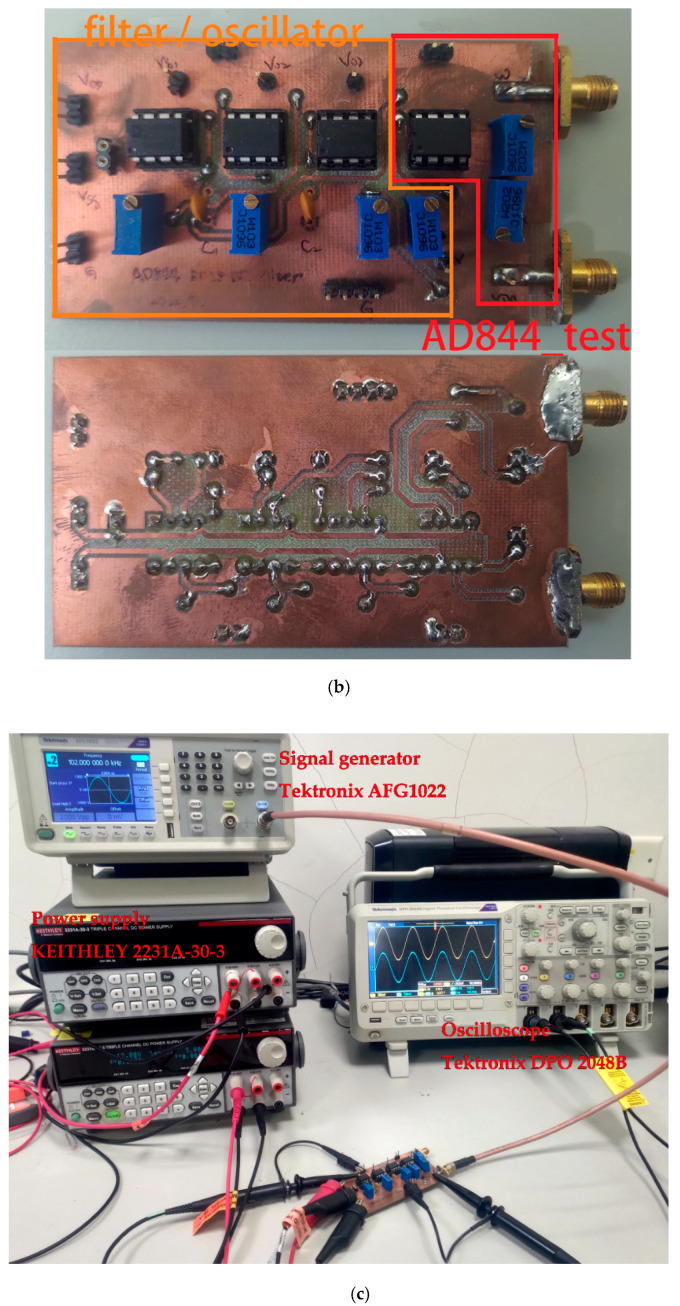

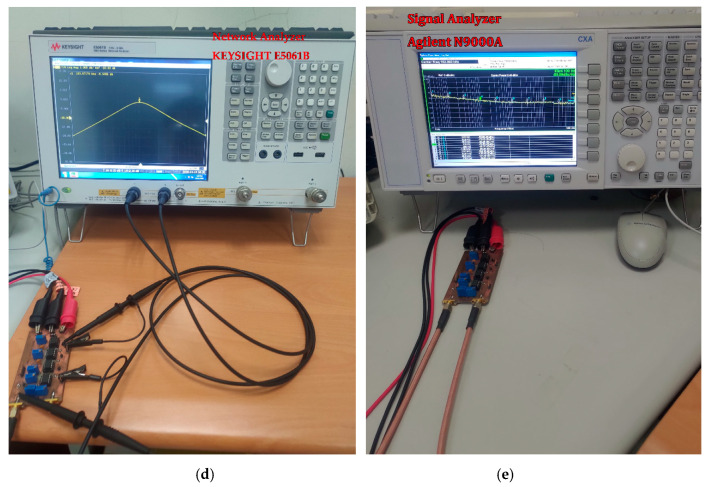
Experimental setup. (**a**) Test setup block diagram; (**b**) the top and bottom of the measured prototype; (**c**) Tektronix DPO 2048B oscilloscope for measuring time domain voltage output, Tektronix AFG1022 signal generator for generating input signal, and Keithley 2231A-30-3 for power supply; (**d**) Keysight E5061B-3L5 network analyzer for measuring frequency domain filter responses; and (**e**) Keysight-Agilent N9000A CXA signal analyzer for measuring filter and oscillator output spectrum.

In order to use the commercial AD844AN IC to prove the real behavior of the proposed VM multifunction biquad filter and fully-uncoupled QO, an experimental test bench was developed, as shown in Figure 5. In Figure 5, the experimental setup uses a printed circuit board (PCB), DC power supply voltage, signal generator, oscilloscope, network analyzer, and signal analyzer. Keithley 2231A-30-3 power supply provides DC power supply voltage to PCB. The time domain of the filter and the output voltage swing of the oscillator are measured by the Tektronix DPO 2048B oscilloscope, and a Tektronix AFG1022 signal generator is used to generate the input signal of the filter. The frequency domain of biquad filter responses is measured by Keysight E5061B-3L5 network analyzer. One dB power gain compression point (P1dB), intermodulation distortion (IMD), phase noise, and output frequency spectrum are measured by the Keysight-Agilent N9000A CXA signal analyzer.

### 3.2. Effective Frequency Ranges of AD844AN-Based Circuit

**Figure 6:**
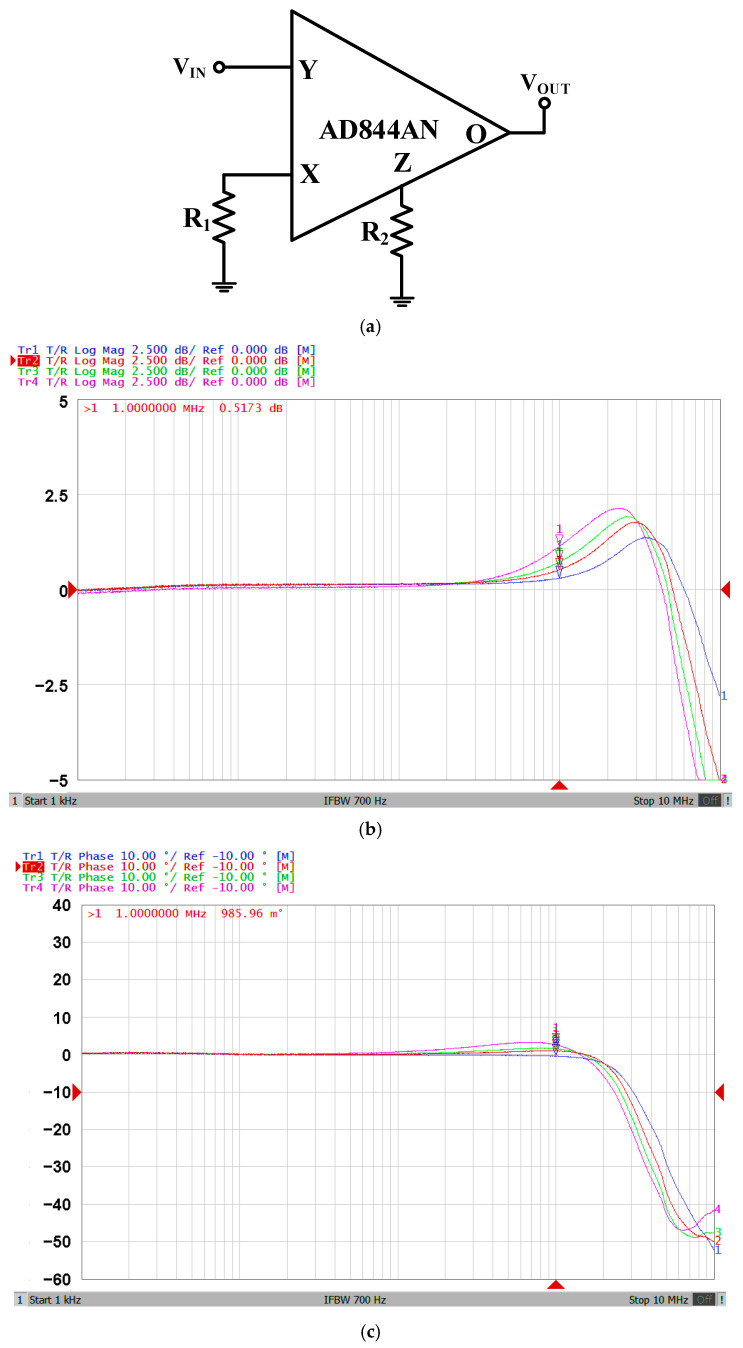
Experimental evidence of the frequency ranges of AD844AN with two equivalent resistances (R = 2 kΩ—blue line; R = 4 kΩ—red line; R = 6 kΩ—green line; and R = 10 kΩ—purple line). (**a**) Circuit diagram for testing gain and phase response frequency ranges of AD844AN; (**b**) measured gain responses of AD844AN; and (**c**) measured gain responses of AD844AN.

The proposed VM multifunction biquad filter and fully-uncoupled QO circuits are simulated by Cadence OrCAD PSpice version 16.6 software. The model parameters of CFOA come from the built-in library AD844/AD. Programming using Intel Core i5-8400 CPU and MATLAB version 2019a has confirmed the effectiveness of simulation and theoretical analysis. For the experiments, the proposed CFOA-based VM multifunction biquad filter and fully-uncoupled QO circuits use AD844AN ICs. The supply voltages for simulation and experiment are ±6 V. In general, the applicability of such filters and oscillators based on CFOA circuits and using AD844AN ICs is usually limited to a few hundred kilohertz [31,32]. To test the frequency ranges of AD844AN, the test circuit based on AD844AN is shown in Figure 6. The supply voltages are ±6 V. In Figure 6a, the selected resistance values are R_1_ = R_2_ = R = 2 kΩ (4 kΩ, 6 kΩ, 10 kΩ). Figure 6b shows the measured gain responses of the AD844AN characteristics. Figure 6c shows the measured phase responses of the AD844AN characteristics. As shown in Figure 6, the frequency range of the AD844AN-based circuit is limited to 1 MHz.

### 3.3. Proposed CFOA-Based VM Multifunction Biquad Filter

**Figure 7:**
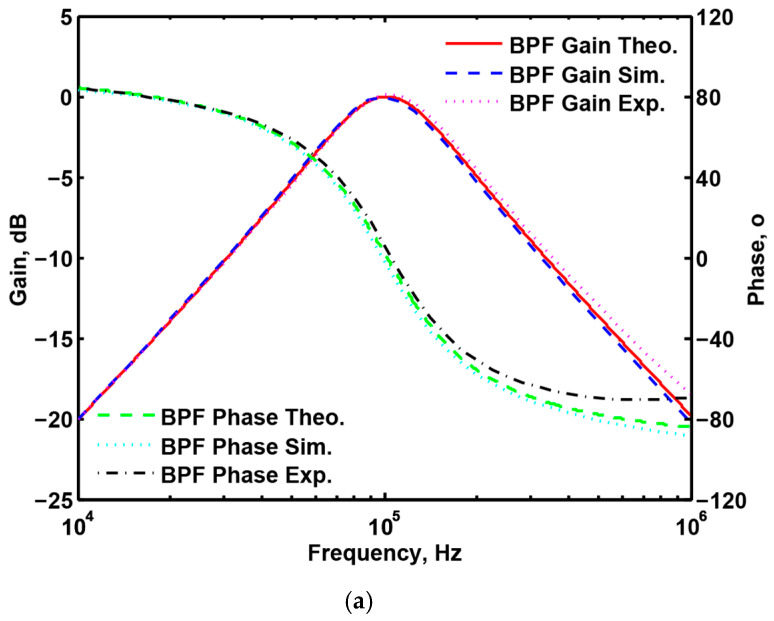

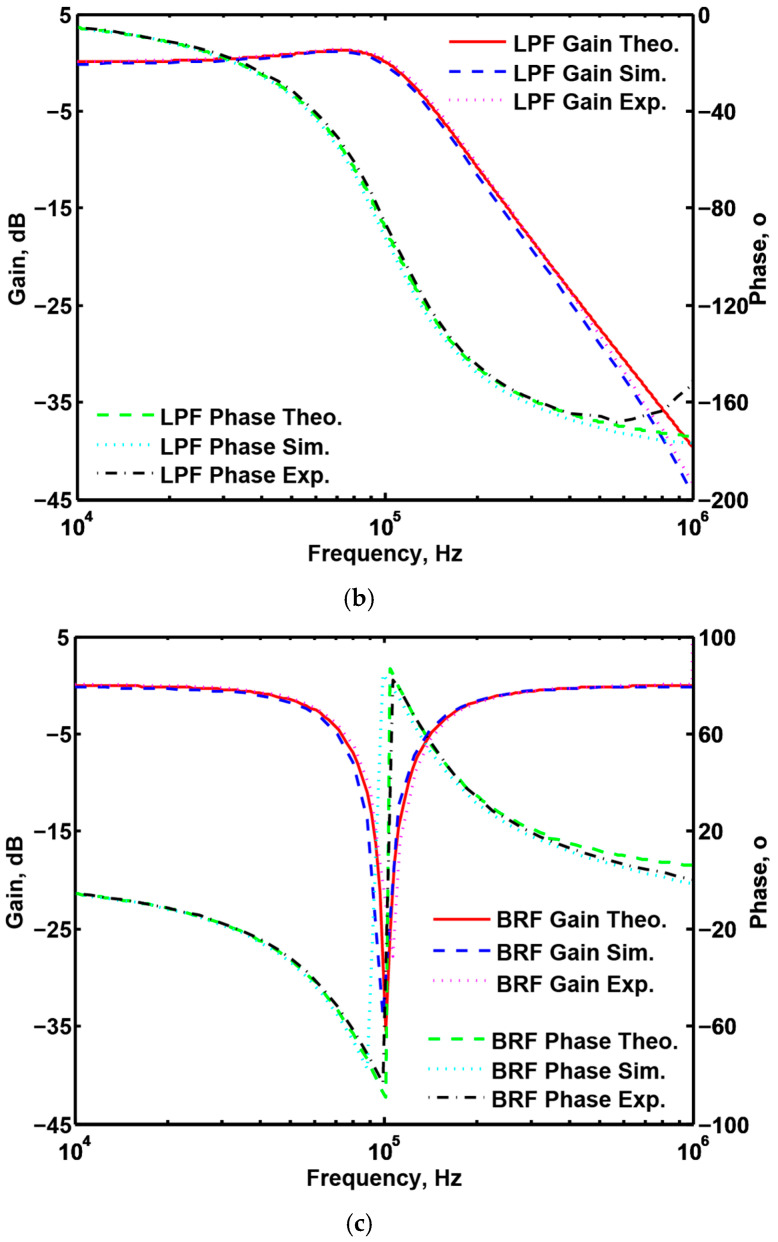
Simulated and experimental filter gain and phase responses with the theory responses. (**a**) The band-pass filter response; (**b**) the low-pass filter response; and (**c**) the band-reject filter response.

**Figure 8:**
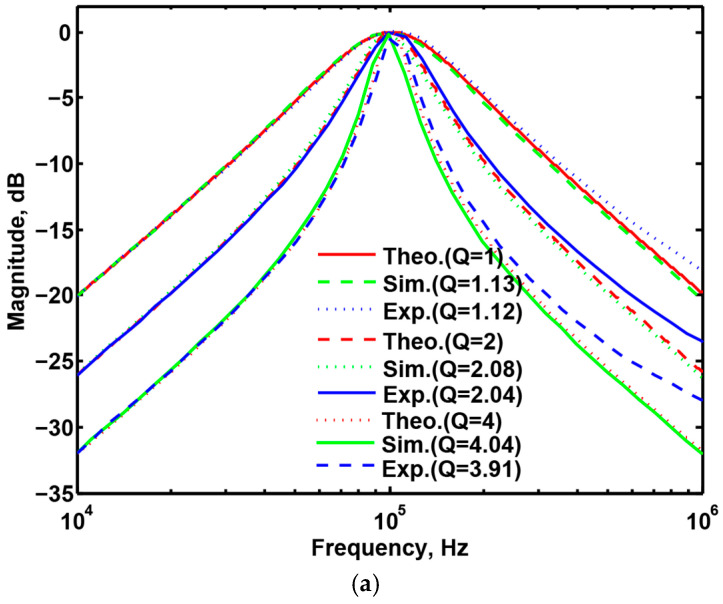

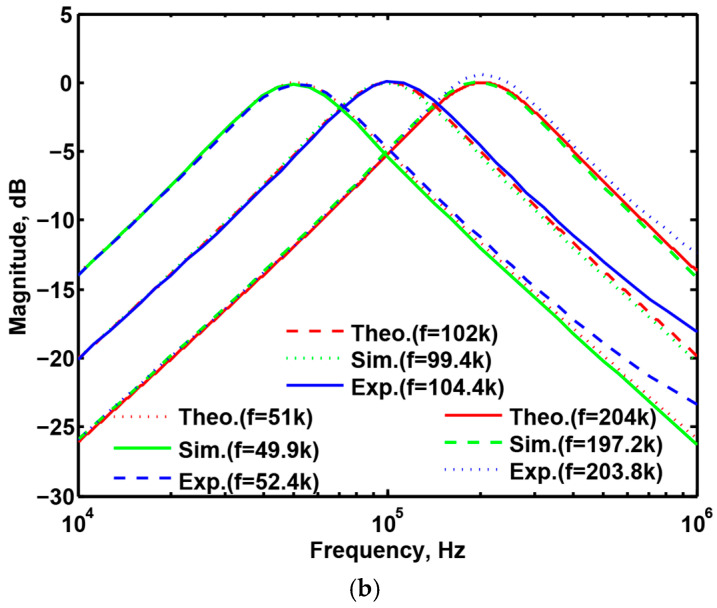
Theoretical, simulated, and experimental gain response of the band-pass filter responses. (**a**) Variation in Q while keeping f_o_; and (**b**) variation in f_o_ while keeping Q.

**Figure 9:**
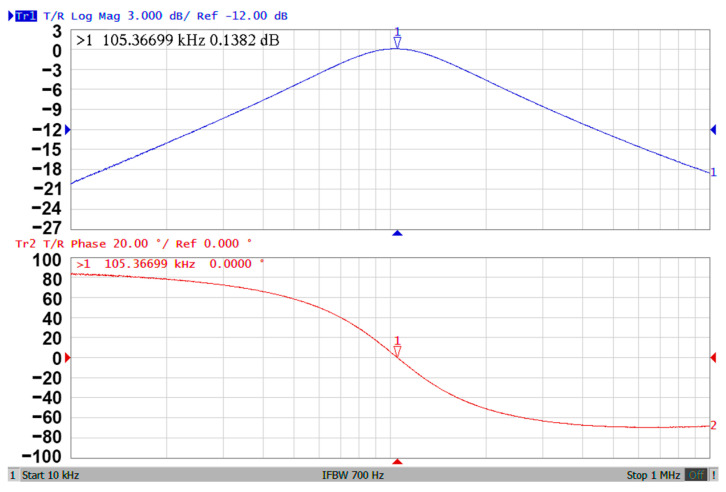
The experimental results of gain and phase responses of the BPF in Figure 2.

**Figure 10:**
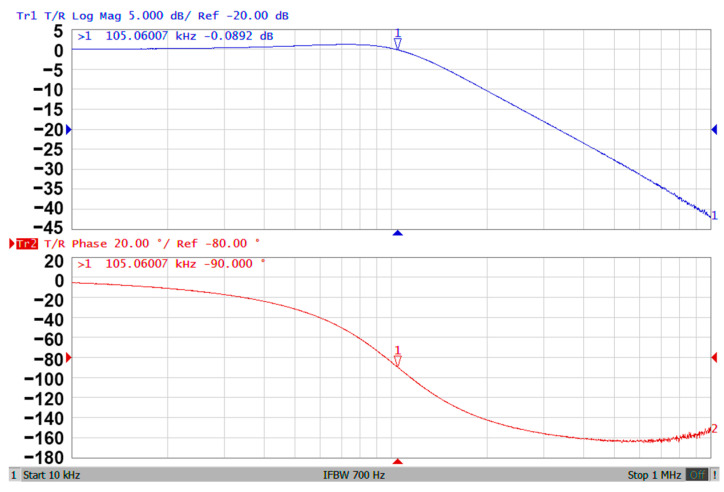
The experimental results of gain and phase responses of the LPF in Figure 2.

**Figure 11:**
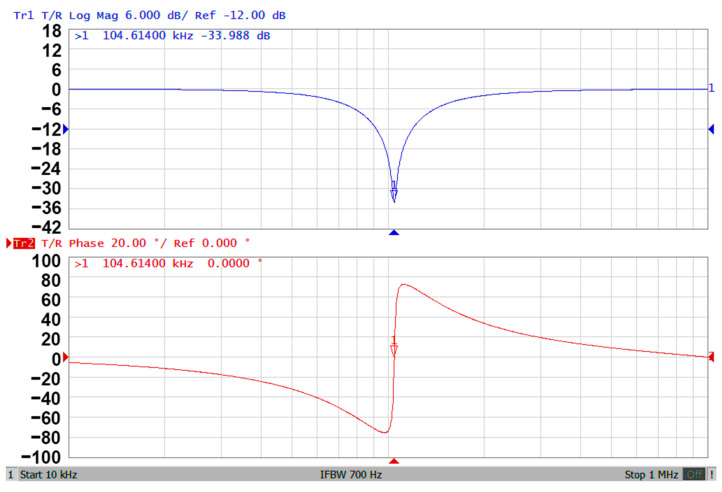
The experimental results of gain and phase responses of the BRF in Figure 2.

**Figure 12:**
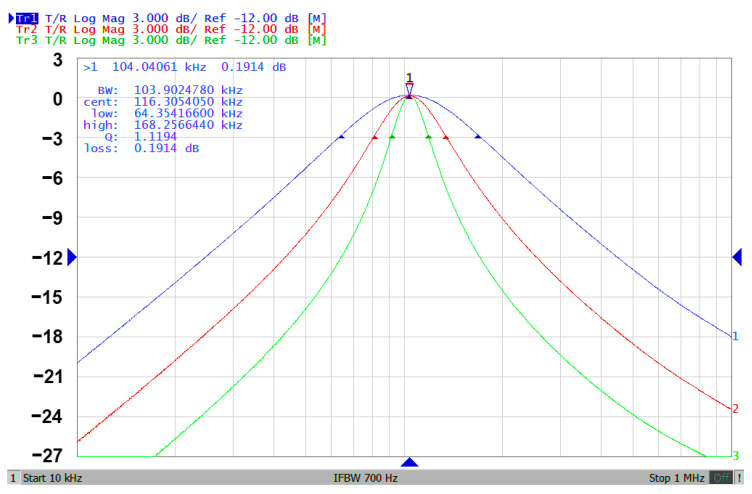
The experimental results of gain responses by varying Q while keeping f_o_ (Q = 1.12—blue line; Q = 2.04—red line; and Q = 3.91—green line).

**Figure 13:**
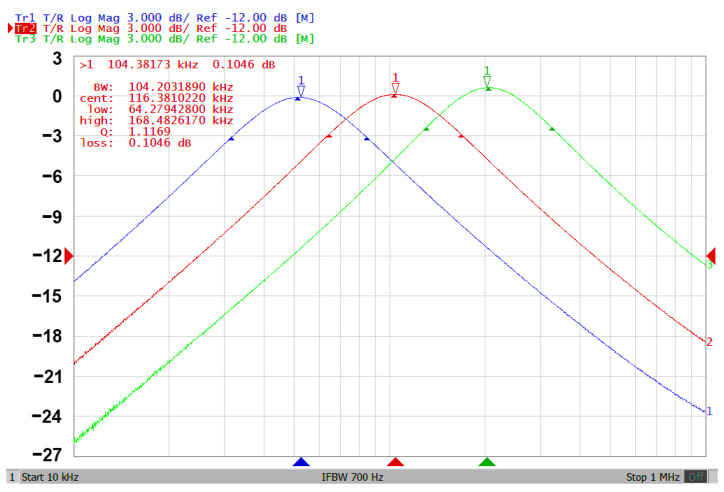
The experimental results of gain responses by varying f_o_ while keeping Q (f_o_ = 52.4 kHz—blue line; f_o_ = 104.4 kHz—red line; and f_o_ = 203.8 kHz—green line).

To validate the theoretical study of Figure 2, Figure 7a–c show the simulation and experimental results of the BPF, LPF and BRF with the theory responses, respectively. In Figure 7, the values of passive elements are selected as C_1_ = C_2_ = 390 pF and R_1_ = R_2_ = R_3_ = R_4_ = 4 kΩ. The selection of these values is to obtain a VM multifunction biquad filter with a center frequency of f_o_ = 102 kHz and a quality factor of Q =1. The total power consumption of the simulation and experimental results is about 255 mW and 168 mW, respectively. By keeping the values of C_1_ = C_2_ = 390 pF, R_1_ = R_2_ = R_3_ = 4 kΩ, the control factor parameter Q of the characteristic filter can be tuned without disturbing f_o_. When the value of R_4_ changes between 16 kΩ, 8 kΩ, and 4 kΩ, this resulted in BPF responses are shown in Figure 8a. Similarly, by keeping the values of C_1_ = C_2_ = 390 pF, R_3_ = R_4_ = 4 kΩ, the control factor parameter f_o_ of the characteristic filter can be tuned without disturbing Q. When Q = 1, and R_1_ and R_2_ are varied between 8 kΩ, 4 kΩ, and 2 kΩ, this resulted in BPF responses are shown in Figure 8b. This range is dependent on the bandwidth of AD844AN. Figure 8a,b show the CFOA-based multifunction biquad filter, whose filter control factor parameters Q and f_o_ have independent tuning capabilities. Figure 9, Figure 10 and Figure 11 show the measured BPF, LPF and BRF responses obtained by a network analyzer, respectively. Figure 12 and Figure 13 show the measured gain responses of Q and f_o_ respectively, as explained in Equation (5). In order to compare the theoretical analysis, the authors derived the measurement data of Figure 9, Figure 10, Figure 11, Figure 12 and Figure 13 and added them as additional traces to Figure 7a–c and Figure 8a,b, respectively. As can be seen, the simulation and experimental results are consistent with the theoretical values. However, the real active components have non-ideal characteristics, such as the parasitic impedance effect of AD844AN, the non-ideal characteristics caused by the frequency dependence of the internal voltage and current transmission of the AD844AN, and the parasitic impedance effect of PCB layout issue. These additional parasitic resistances and capacitances of the AD844AN, the PCB layout issue, and the tolerances of the working resistors and capacitors will have main effects on circuit accuracy. 

**Figure 14:**
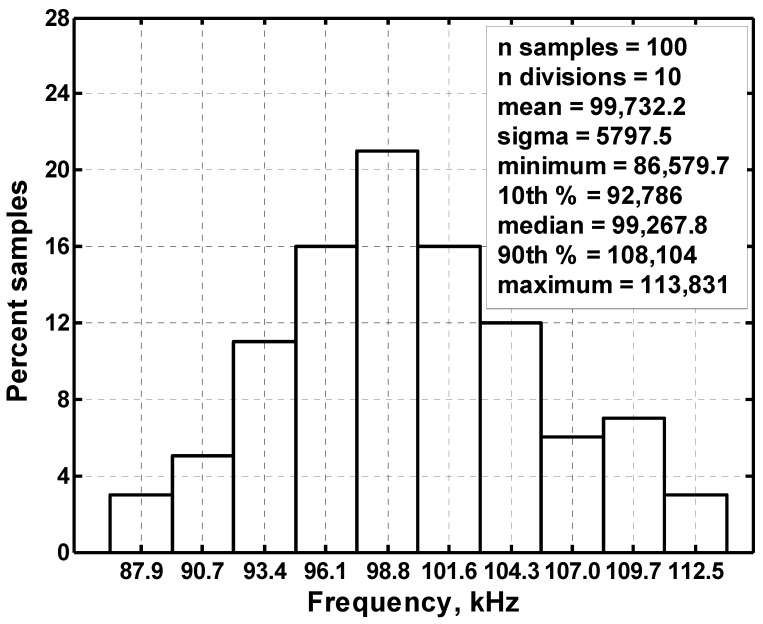
Histogram of the Monte-Carlo analysis.

The Monte Carlo analysis method is used to study the VM CFOA-based multifunction biquad filter. Statistical analysis of Monte Carlo 100 simulation can be performed. Figure 14 shows the histogram of center frequency obtained from the Monte-Carlo analysis of the BPF response in Figure 2. The resistor and capacitor values in Figure 2 are chosen as 4 kΩ and 390 pF, respectively, and the Gaussian variation of the resistance and capacitance is 5%. According to Monte-Carlo simulation, the center frequency varies between 86.6 and 113.8 kHz and the f_o_ value of the BPF response is affected in the range of −15.1% to 11.6%.

**Figure 15:**
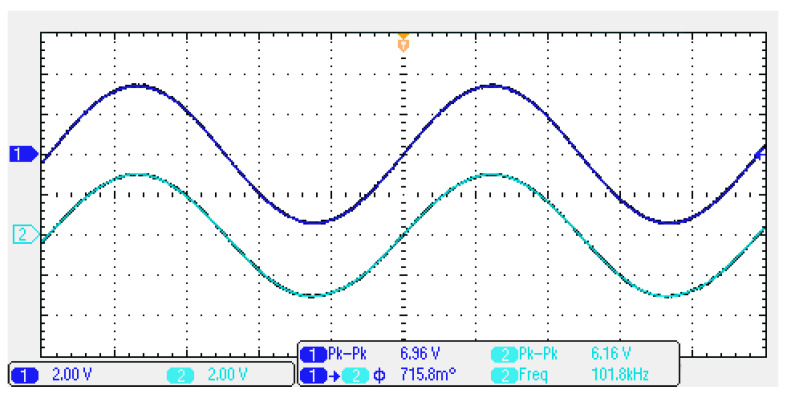
The experimental results of input (channel 1, blue line) and output (channel 2, cyan line) time-domain experimental voltage waveforms.

**Figure 16:**
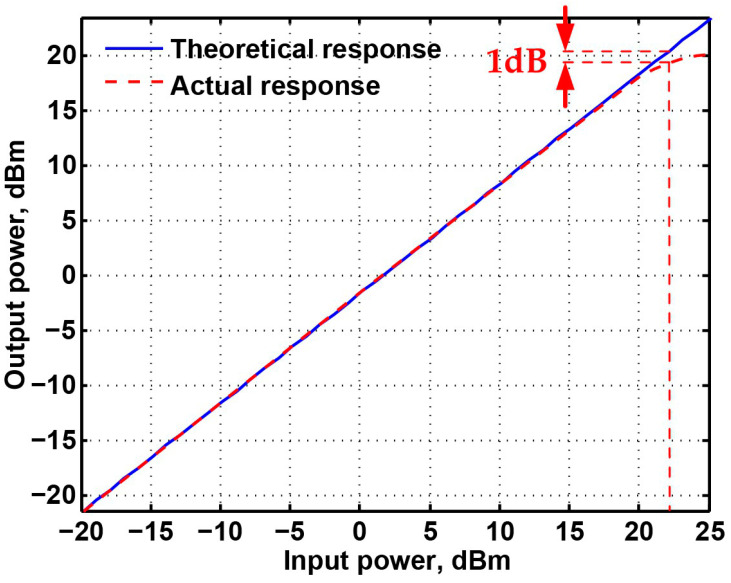
The simulation and measurement P1dB of the BPF with input power at the output voltage V_o1_.

To obtain the input dynamic range of the proposed CFOA-based VM multifunction biquad filter in Figure 2, the experiment was repeated for a sinusoidal input signal of 102 kHz to show the filter operation in time-domain. Figure 15 shows the input and output voltage waveforms of the experimental BPF. The waveform shows that the amplitude is 6.96 V_pp_ without signification distortion. The measured center frequency is about 101.8 kHz, which is close to theoretical value of 102 kHz with −0.2% error rate. To illustrate the linear range of the circuit, P1dB is an important parameter for evaluating linear range of the circuit, when the output is saturated in the circuit. This is defined as the input power that results in the circuit gain to decrease by 1 dB. Input and output power gain is the relationship of output power = input power + gain. To evaluate the linear range of the VM biquad filter, consider the BPF, LPF and BRF in Equations (1) to (3). Figure 16 shows the measured P1dB of the BPF at an input power with a center frequency of 102 kHz. The P1dB of the BPF measured at V_o1_ is about 22 dBm with respect to input power. The measured linearity performance of P1dB and total power consumption with different input signal voltages are summarized in Table 3. It should be noted that Table 3 is the measurement results of spectrum analysis, whose input impedance is 50 Ω, so the measurement result of output power has slightly attenuated. The parasitic resistances and capacitances of the AD844AN, the PCB layout issue, and the tolerances of the working resistors and capacitors will also have effects on circuit accuracy.

**Figure 17:**
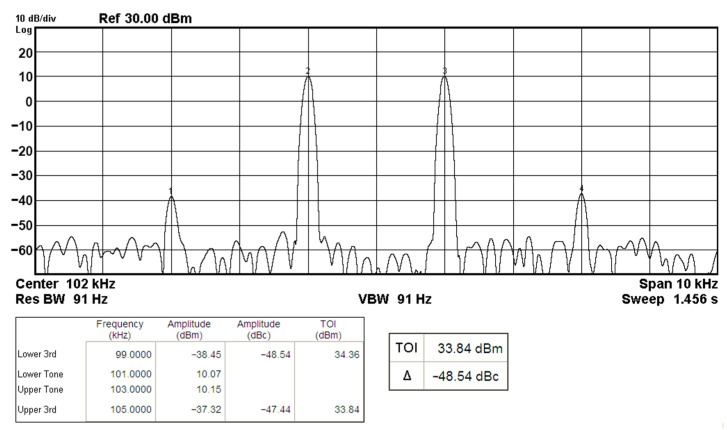
The measured BPF output spectrum for a two-tone intermodulation distortion test.

To represent the nonlinearity of the proposed VM multifunction biquad filter proposed in Figure 2, the two-tone test of IMD has been used to characterize the nonlinearity of BPF response. Figure 17 shows the frequency spectrum analysis of BPF through intermodulation characteristics by applying two-tone signals, f_1_ and f_2_, around the corner frequency of f_o_ = 102 kHz. In Figure 17, a low-frequency tone of f_1_ = 101 kHz and a high-frequency tone of f_2_ = 103 kHz are used with equal input amplitudes of 4.5 V_pp_. As shown in Figure 17, the measured value of the third-order IMD is around −48.54 dBc, and the third-order intercept (TOI) point is around 33.84 dBm.

### 3.4. Proposed CFOA-Based VM QO Experimental Results

**Figure 18:**
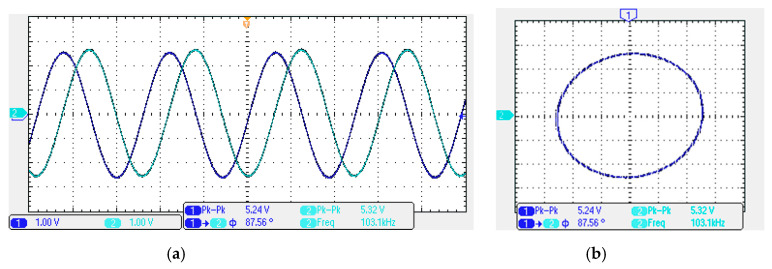
The experimental results. (**a**) The quadrature voltage outputs V_o1_ (blue) and V_o2_ (cyan); and (**b**) X-Y pattern.

**Figure 19:**
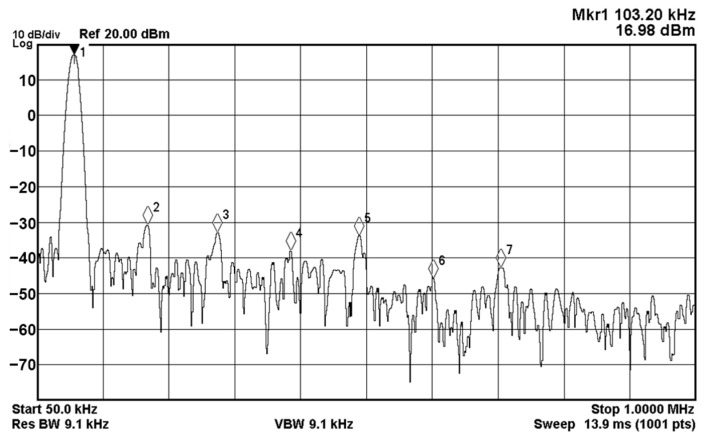
The measured output frequency spectrum of V_o1_.

**Figure 20:**
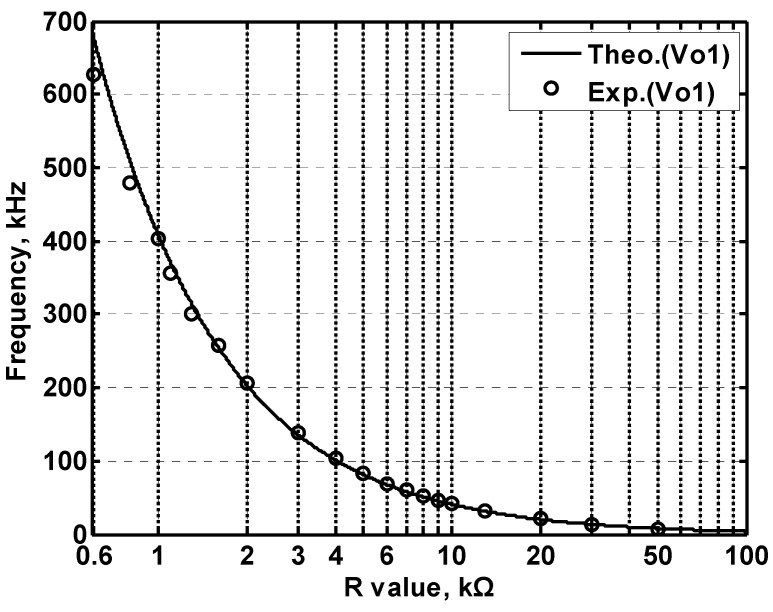
The measured oscillation frequency with simultaneously varying the values of R_1_ and R_2_ for the proposed VM quadrature oscillator in Figure 4.

**Figure 21:**
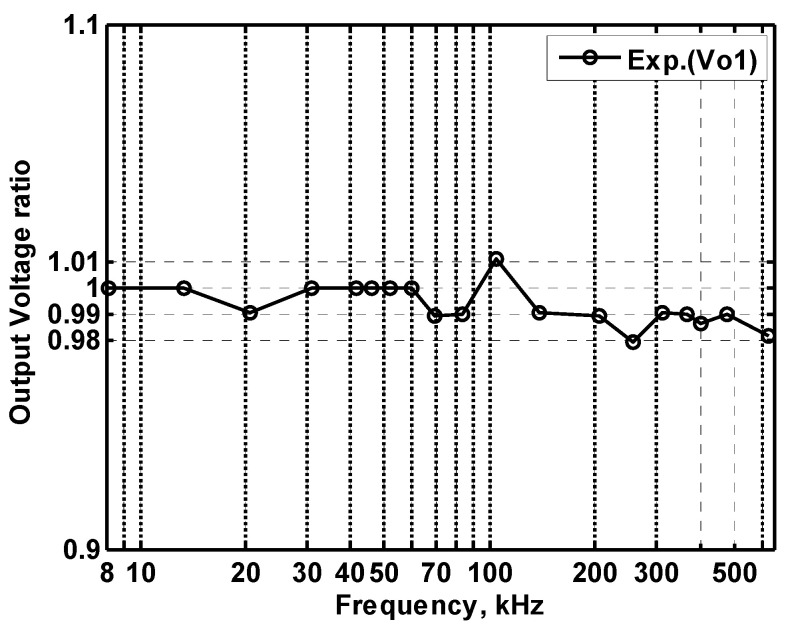
The measured relationship between the oscillation frequency and the magnitude ratio of quadrature output voltages.

**Figure 22:**
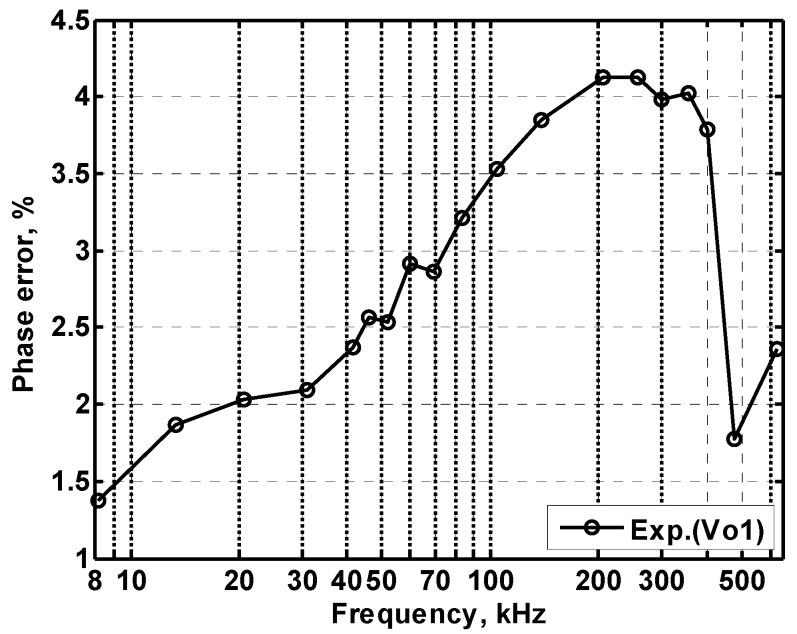
The measured phase error of oscillation frequency.

**Figure 23:**
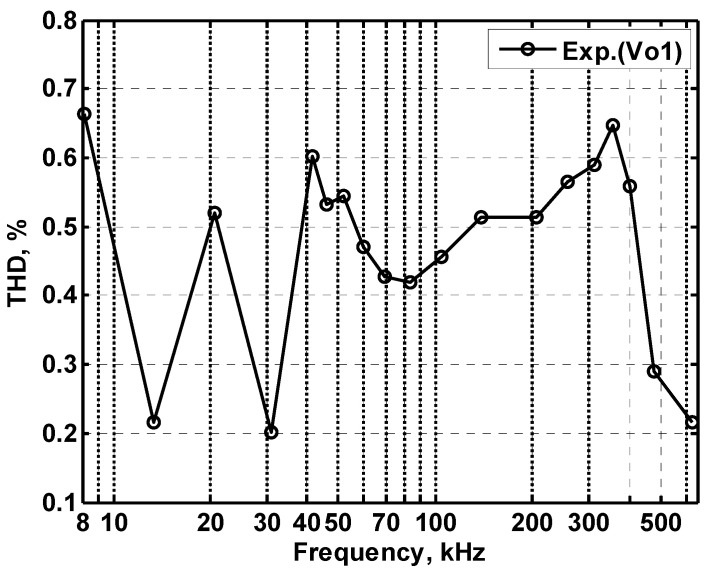
The measured THD of oscillation frequency.

In order to get the theoretical oscillation frequency f_o_ = 102 kHz of the VM fully-uncoupled QO proposed in Figure 4, the values of the passive components are selected as R_1_ = R_2_ = R_3_ = 4 kΩ, and C_1_ = C_2_ = 390 pF, and R_4_ = 4.02 kΩ is greater than R_3_ to ensure that the oscillations starts. Figure 18a shows the output waveforms of V_o1_ and V_o2_. Figure 18b shows V_o1_ versus V_o2_ in the X-Y plot. Figure 19 shows the frequency spectrum analysis of the oscillator output voltage. From Figure 18 and Figure 19, the measured oscillation frequency is 103.2 kHz, which is closed to theoretical result of 102 kHz. The error percentage between the theoretical oscillation frequency and measured oscillation frequency is 1.08%. Total harmonic distortion (THD) is about 0.63%. Figure 20 shows the measured value of the oscillation frequency of Figure 4, which is measured by simultaneously changing the values of resistors R_1_ and R_2_. According to Figure 20, the experimental oscillation frequency varies from 8.16 to 628 kHz. These measurement results are close to theoretical prediction and confirm the feasibility of the proposed VM QO. Figure 21 shows the measured oscillation frequency relative to the magnitude ratio of the two output voltages, as explained in Equation (16). As can be seen, the constant amplitude ratio of two quadrature sinusoidal waveforms can be realized when tuning FO. The magnitude ratio of the two output voltages fluctuates from −2.04% to 1.14%. Automatic level control can obtain better oscillation amplitude [34]. Figure 22 shows the measured phase error percentage of the quadrature voltage outputs. The VM QO operates at frequencies from 8.16 to 628 kHz, and the maximum deviation from 90° is less than 4.12%. The THD of the quadrature output voltage waveforms is shown in Figure 23. It was found that the measured THD percentage fluctuated between 0.2% and 0.66% when VM QO was operating in the frequency range of 8.16 to 628 kHz. Obviously, the experimental results are consistent with the theoretical values. However, the real active components have the non-ideal characteristics of AD844AN parasitic impedance effect and PCB layout issue. According to AD844AN datasheet [29], the actual AD844AN exhibits a non-zero input resistance of R_IN_ = 50 Ω at the port -IN. The parallel combination of R_Z_ and C_Z_ is the parasitic impedances connected from the current output port T_Z_ of the AD844AN. For AD844AN, the nominal values are R_Z_ = 3 MΩ and C_Z_ = 4.5 pF. These additional parasitic resistances and capacitances of the AD844AN, the PCB layout issue, and the tolerances of the working resistors and capacitors will have main effects on circuit accuracy.

**Figure 24:**
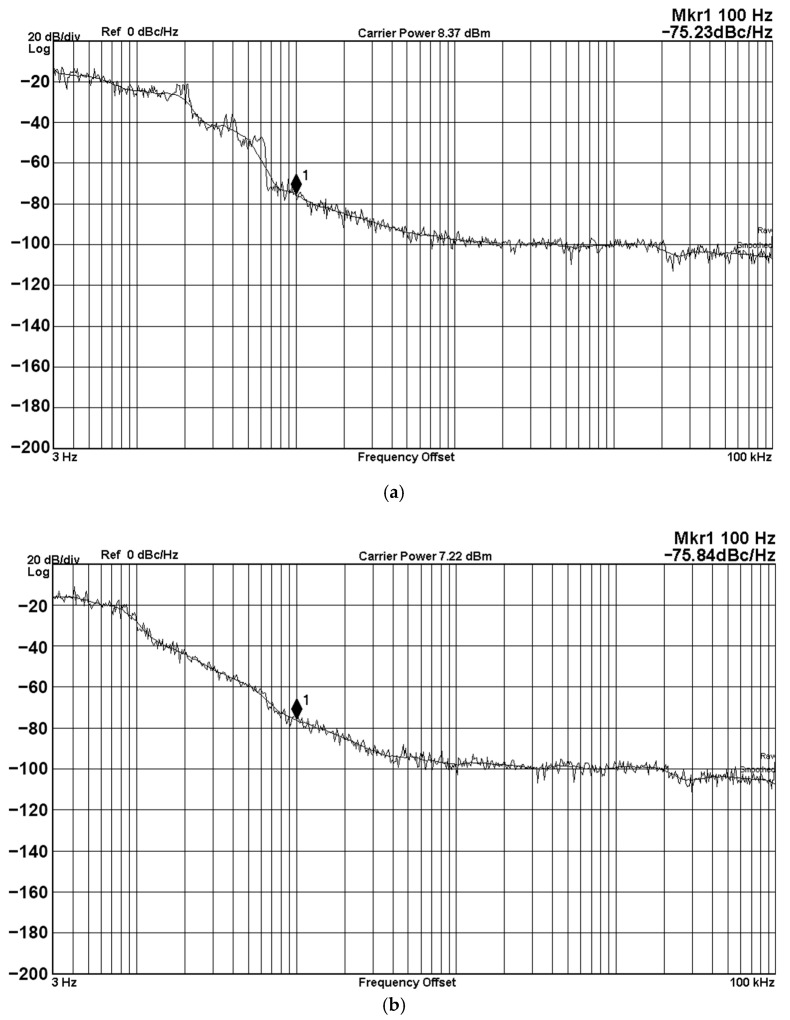
Phase noise performance with different frequency offsets measured from 3 Hz offset frequency. (**a**) V_o1_ output phase noise; and (**b**) V_o2_ output phase noise.

The noise of the oscillator will affect the characteristics of frequency spectrum and timing. Even if a small amount of noise can cause large variants in the oscillator’s spectrum and timing. The Keysight-Agilent N9000A CXA signal analyzer provides a phase noise measurement solution starting at 3 Hz. Figure 24 shows the phase noise performance of the operating frequency from 3 Hz to 100 kHz with different frequency offsets. The phase noise measurement result in Figure 24 shows that the phase noise of the proposed VM QO is less than −75.23 dBc/Hz at a 100 Hz offset, which has little impact on the frequency spectrum and timing. Another interesting parameter is the phase noise FoM of the proposed VM fully-uncoupled QO. Table 4 lists the phase noise performance measured at different oscillator frequency at an offset frequency of 100 Hz with a supply voltages of ±6 V and a power consumption of 300 mW. According to Equation (17), and using the data in Table 4, the minimum phase noise FoMs of V_o1_ and V_o2_ are 90.3 dBc/Hz and 100.4 dBc/Hz, respectively. The measured phase noise performance under different oscillator supply voltages are summarized in Table 5. 

## 4. Conclusions

The paper proposes an independently tunable VM multifunction biquad filter with high-input impedance and a VM fully-uncoupled QO realized by the proposed VM multifunction biquad filter. Both proposed circuits use three CFOAs as active components, while using four resistors and two grounded capacitors as passive components. The proposed VM multifunction biquad filter offers the following important features: (i) simultaneous realization of BRF, BPF, and LPF voltage transfer functions without component value constraints, (ii) high-input impedance, (iii) using only two grounded capacitors, (iv) the X ports of the CFOAs are connected directly to the resistors, (v) independent controllability of the biquad filter control factor parameters ω_o_ and Q, and (vi) fully-uncoupled adjustable of CO and FO when transformed to VM QO. Unlike the recently reported VM CFOA-based multifunction biquad filter [27], the attractive feature of the proposed VM multifunction biquad filter is that it can independently control the filter control factor parameters ω_o_ and Q, and due to the characteristics of fully-uncoupled adjustable of CO and FO, it can be converted into VM QO. The proposed VM fully-uncoupled QO improves unbalance of produced quadrature output voltages V_o1_ and V_o2_, and solves the amplitude instability. The constant amplitude ratio of two quadrature sinusoidal waveforms can be realized when tuning FO. The QO oscillation frequency could be tuned in the range of 8.16 to 628 kHz and tested with ±6 V voltage power supplies. The minimum phase noise FoM in the entire tuning range is 90.3 dBc/Hz at 100 Hz offset frequency. The measured THD is less than 0.7%. The measured P1dB and IMD of VM biquad filter are 22 and 33.84 dBm, respectively. PSpice simulations and experimental results based on commercial IC AD844AN are used to verify the theoretical characteristics of the proposed CFOA-based circuits.

## Figures and Tables

**Table 1:** Comparison of the previous reported CFOA-based VM biquad filters.

Parameter	(i)	(ii)	(iii)	(iv)	(v)	(vi)	(vii)	Simul./Meas.	Supply (V)	Technology
Ref. [12]	yes	no	no	no	yes	no	no	Meas.	N/A	AD844 ICs
Ref. [13]	yes	no	no	no	yes	no	no	Meas.	N/A	AD844 ICs
Ref. [14]	yes	no	no	no	yes	no	no	Simul.	±12	AD844 model
Ref. [15]	yes	no	yes	no	yes	no	no	Simul.	N/A	AD844 model
Ref. [16]	yes	no	no	no	yes	no	no	Simul.	N/A	AD844 model
Ref. [17]	no	yes	yes	no	yes	no	no	Both	±12	AD844 ICs
Ref. [18]	yes	no	no	no	yes	no	no	Meas.	N/A	AD844 ICs
Ref. [19]	yes	yes	yes	no	yes	no	no	Meas.	N/A	AD844 ICs
Ref. [20]	yes	no	no	no	no	no	no	Simul.	N/A	AD844 model
Ref. [21]	no	yes	no	yes	yes	no	no	Meas.	±5	AD844 ICs
Ref. [22]	yes	yes	no	yes	yes	no	no	Simul.	±5	AD844 model
Ref. [23]	no	yes	yes	yes	yes	no	no	Simul.	N/A	AD844 model
Ref. [24]	yes	no	yes	yes	no	no	no	Simul.	N/A	AD844 model
Ref. [25]	no	yes	yes	yes	yes	no	no	Both	N/A	AD844 ICs
Ref. [26]	yes	yes	yes	yes	no	no	no	Simul.	±12	AD844 model
Ref. [27]	yes	yes	yes	yes	yes	no	no	Both	±6	AD844 ICs
Proposed	yes	yes	yes	yes	yes	yes	yes	Both	±6	AD844 ICs

Note: (i) Use up to three CFOAs; (ii) use only two grounded capacitors; (iii) realize three standard filter transfer functions simultaneously; (iv) high-input impedance; (v) CFOA’s X ports input parasitic resistances can be easily accommodated in an external resistors; (vi) independent control of the filter control factor parameters pole frequency and quality factor; (vii) transformed into a voltage-mode QO with fully-uncoupled adjustable of the condition of oscillation and the frequency of oscillation; Simul.: simulation result; Meas.: measurement result; ICs: integrated circuits; N/A—not available or not tested.

**Table 2:** Characteristic comparisons with recent previous study.

Parameter	Ref. [27]	Proposed
Number of active and passive components of the biquad filter	3 CFOAs, 3 R, 2 C	3 CFOAs, 4 R, 2 C
Number of active and passive components of the quadrature oscillator	3 CFOAs, 4 R, 2 C	3 CFOAs, 4 R, 2 C
Center frequency of the biquad filter (kHz)	39.79	102
Independent tuning of the filter control factor parameters ω_o_ and Q	no	yes
Fully-uncoupled tuning of the oscillator parameters CO and FO	no	yes
Constant amplitude ratio of quadrature waveforms	no	yes
Measured the oscillation frequency range (kHz)	N/A	8.16~628
Measured the total harmonic distortion of the quadrature oscillator (%)	N/A	<0.7
Measured the power dissipation (mW)	180	168
Measured the input one-dB power gain compression point (dBm)	12	22
Measured the third-order intermodulation distortion point (dBm)	21.59	33.84

**Table 3:** The linearity performance of P1dB and total power consumption with different input signal voltages.

Supply (V)	Band-Pass Filter (V_o1_)				Low-Pass Filter (V_o2_)				Band-Reject Filter (V_o3_)			
	Power (dBm)		P_D_ (mW)	P1dB (dBm)	Power (dBm)		P_D_ (mW)	P1dB (dBm)	Power(dBm)		P_D_ (mW)	P1dB (dBm)
	Input	Output			Input	Output			Input	Output		
±6	−20	−21.56	264	22	−20	−21.38	264	21	−20	−21.19	264	22
	−10	−11.53	264		−10	−11.32	264		−10	−11.15	264	
	0	−1.49	264		0	−1.26	264		0	−1.1	264	
	10	8.46	300		10	8.67	300		10	9	300	
	20	18.16	396		20	18.15	396		20	18.72	408	
	21	18.91	408		21	18.79	408		21	19.42	432	
	22	19.46	420		22	19.25	408		22	19.82	444	
	23	19.8	432		23	19.47	420		23	20.11	456	
	24	20.02	432		24	19.58	420		24	20.33	456	
	25	20.24	444		25	19.65	420		25	20.52	456	
±9	−20	−21.6	396	26.8	−20	−21.3	396	26.1	−20	−21.23	396	26.3
	−10	−11.56	396		−10	−11.26	396		−10	−11.19	396	
	0	−1.5	396		0	−1.22	396		0	−1.12	396	
	10	8.44	450		10	8.68	450		10	8.97	450	
	20	18.35	666		20	18.4	666		20	19.02	684	
	24	22.26	846		24	22.2	846		24	22.92	864	
	25	23.18	900		25	23.09	900		25	23.76	918	
	26	23.92	936		26	23.77	936		26	24.23	972	
	26.8	24.2	972		26.1	23.8	936		26.3	24.3	972	
	27	24.28	990		27	24.11	972		27	24.51	990	
±12	−20	−21.54	528	28.5	−20	−21.26	528	27.7	−20	−21.15	528	27.5
	−10	−11.5	528		−10	−11.23	528		−10	−11.11	528	
	0	−1.46	528		0	−1.22	528		0	−1.06	528	
	10	8.49	600		10	8.7	600		10	9.04	600	
	20	18.35	888		20	18.46	888		20	19.04	912	
	25	23.3	1224		25	23.2	1224		25	24.08	1272	
	26	24.26	1320		26	24.03	1320		26	24.92	1368	
	27	25.12	1416		27	25.1	1416		27	25.35	1440	
	28	25.8	1608		27.7	25.58	1536		27.5	25.5	1464	
	28.5	26	1680		28	25.7	1584		28	25.72	1512	
±15	−20	−21.48	660	28.8	−20	−21.17	660	28	−20	−21.8	630	27.5
	−10	−11.36	660		−10	−11.14	660		−10	−11.04	630	
	0	−1.41	690		0	−1.1	690		0	−0.99	660	
	10	8.53	750		10	8.82	750		10	9.1	750	
	20	18.43	1110		20	18.57	1110		20	19.11	1110	
	25	23.35	1560		25	23.34	1560		25	23.98	1590	
	26	24.33	1680		26	24.36	1680		26	24.63	1710	
	27	25.15	1920		27	25.16	1830		27	25.4	1800	
	28	25.9	2010		28	25.8	2010		27.5	25.7	1830	
	28.8	26.4	2160		28.2	25.9	2040		28	25.8	1890	

Note: P_D_: Dynamic power consumption; P1dB: input one-dB power gain compression point.

**Table 4:** Phase noise performance measured at different oscillator frequencies.

Quadrature Output Voltage V_o1_			Quadrature Output Voltage V_o2_		
f_o_ (kHz)	Phase Noise (dBc/Hz)	FoM (dBc/Hz)	f_o_ (kHz)	Phase Noise (dBc/Hz)	FoM (dBc/Hz)
8.1	85.23	98.5	8.1	87.6	101.2
13.3	83.36	103.9	13.3	84.35	102.2
20.7	84.27	105.7	20.7	80.59	102.3
30.9	84.98	110.0	30.9	84.61	109.8
41.6	83.56	111.2	41.6	81.87	109.7
46.1	84.09	112.4	46.1	84.39	113.1
52.1	82.84	112.4	52.1	82.66	112.4
59.6	81.95	112.7	59.6	83.29	114.2
69.3	80.74	112.8	69.3	81.76	114.0
83.4	92.25	126.5	83.4	82.21	116.0
104.1	75.23	110.8	104.1	74.24	110.0
138	68.28	120.8	138	65.43	103.6
205.8	62.26	118.4	205.8	76.57	118.2
256.7	47.09	90.3	256.7	76.48	120.1
300.5	75.29	119.9	300.5	71.3	116.3
356.9	73.92	119.9	356.9	53.93	100.4
402.7	73.54	120.7	402.7	72.43	119.9

Note: f_o_: Oscillation frequency; and FoM: Figure-of-Merit.

**Table 5:** Phase noise performance measured at different supply voltages.

V_DC_	Output Voltage V_o1_					Output Voltage V_o2_				
	Phase Noise (dBc/Hz)	Δf (Hz)	P_DC_ (mW)	f_o_ (kHz)	FoM (dBc/Hz)	Phase Noise (dBc/Hz)	Δf (Hz)	P_DC_ (mW)	f_o_ (kHz)	FoM (dBc/Hz)
±4.5	71.41	100	216	102	108.4	73.46	100	225	102	110.2
±6	72.26	100	300	102	107.8	74.24	100	300	102	109.8
±9	72.67	100	432	102	106.7	76.91	100	450	102	110.7
±12	71.9	100	576	102	104.6	71.6	100	624	102	103.9
±15	69.88	100	720	102	101.7	76.28	100	780	102	107.7

Note: VDC: Supply voltage; Δf: Offset frequency; PDC: Power consumption; fo: Oscillation frequency; and FoM: Figure-of-Merit.

## References

[1] Tran H.D., Wang H.Y., Lin M.C., Nguyen Q.M. (2015). Synthesis of cascadable DDCC-based universal filter using NAM. Appl. Sci..

[2] Herencsar N., Koton J., Hanak P. (2017). Universal voltage conveyor and its novel dual-output fully-cascadable VM APF application. Appl. Sci..

[3] Safari L., Barile G., Ferri G., Stornelli V. (2019). A new low-voltage low-power dual-mode VCII-based SIMO universal filter. Electronics.

[4] Wang S.F., Chen H.P., Ku Y., Lin Y.C. (2019). Versatile tunable voltage-mode biquadratic filter and its application in quadrature oscillator. Sensors.

[5] Wang H.Y., Tran H.D., Nguyen Q.M., Yin L.T., Liu C.Y. (2014). Derivation of oscillators from biquadratic band pass filters using circuit transformations. Appl. Sci..

[6] Sotner R., Jerabek J., Langhammer L., Dvorak J. (2018). Design and analysis of CCII-Based oscillator with amplitude stabilization employing optocouplers for linear voltage control of the output frequency. Electronics.

[7] Ullah F., Liu Y., Li Z., Wang X., Sarfraz M.M., Zhang H. (2018). A bandwidth-enhanced differential LC-voltage controlled oscillator (LC-VCO) and superharmonic coupled quadrature VCO for K-band applications. Electronics.

[8] Márquez A., Pérez-Bailón J., Calvo B., Medrano N., Martínez P.A. (2018). A CMOS self-contained quadrature signal generator for SoC impedance spectroscopy. Sensors.

[9] Jaikla W., Adhan S., Suwanjan P., Kumngern M. (2020). Current/voltage controlled quadrature sinusoidal oscillators for phase sensitive detection using commercially available IC. Sensors.

[10] Wang S.F., Chen H.P., Ku Y., Lee C.L. (2020). Versatile voltage-mode biquadratic filter and quadrature oscillator using four OTAs and two grounded capacitors. Electronics.

[11] Ibrahim M.A., Minaei S., Kuntman H. (2005). A 22.5 MHz current-mode KHN biquad using differential voltage current conveyor and grounded passive element. AEU Int. J. Electron. Commun..

[12] Liu S.I., Wu D.S. (1995). New current-feedback amplifier-based universal biquadratic filter. IEEE Trans. Instrum. Meas..

[13] Horng J.W. (2000). New configuration for realizing universal voltage-mode filter using two current-feedback amplifiers. IEEE Trans. Instrum. Meas..

[14] Tangsrirat W., Surakampontorn W. (2009). Single-resistance-controlled quadrature oscillator and universal biquad filter using CFOAs. AEU Int. J. Electron. Commun..

[15] Shah N.A., Iqbal S.Z., Rather M.F. (2005). Versatile voltage-mode CFA-based universal filter. AEU Int. J. Electron. Commun..

[16] Shan N.A., Rather M.F., Iqbal S.Z. (2005). CFA-based three input and two outputs voltage-mode universal filer. Indian J. Pure Appl. Phy..

[17] Singh V.K., Singh A.K., Bhaskar D.R., Senani R. (2006). New universal biquads employing CFOAs. IEEE Trans. Circuits Syst. II Express Briefs.

[18] Horng J.W. (2013). Voltage-mode universal biquad with five inputs and two outputs using two current feedback amplifiers. Indian J. Eng. Mater. Sci..

[19] Chang C.M., Hwang C.S., Tu S.H. (1994). Voltage-mode notch, lowpass and bandpass filter using current-feedback amplifiers. Electron. Lett..

[20] Shah N.A., Malik M.A. (2005). Multifunction filter using current feedback amplifiers. Frequenz.

[21] Nikoloudis S., Psychalinos C. (2010). Multiple input single output universal biquad filter with current feedback operational amplifiers. Circuits Syst. Signal Process..

[22] Topaloglu S., Sagbas M., Anday F. (2012). Three-input single-output second-order filters using current-feedback amplifiers. AEU Int. J. Electron. Commun..

[23] Singh A.K., Senani R. (2005). CFOA-based state-variable biquad and its high-frequency compensation. IEICE Electron. Express.

[24] Horng J.W., Lee M.H. (1997). High input impedance voltage-mode lowpass, bandpass and highpass filter using current-feedback amplifiers. Electron. Lett..

[25] Shan N.A., Malik M.A. (2005). High input impedance voltage-mode lowpass, bandpass, highpass and notch filter using current feedback amplifiers. Indian J. Eng. Mater. Sci..

[26] Shan N.A., Malik M.A. (2005). New high input impedance voltage-mode lowpass, bandpass and highpass filter using current feedback amplifiers. J. Circuits Syst. Comp..

[27] Wang S.F., Chen H.P., Ku Y., Chen P.Y. (2019). A CFOA-based voltage-mode multifunction biquadratic filter and a quadrature oscillator using the CFOA-based biquadratic filter. Appl. Sci..

[28] Soliman A.M. (1996). Applications of the current feedback operational amplifiers. Analog Integr. Circuits Process..

[29] (2017). AD844: 60 MHz, 2000 V/μs, Monolithic Op Amp with Quad Low Noise Data Sheet (Rev. G). www.linear.com.

[30] Bhaskar D.R., Gupta S.S., Senani R. (2012). New CFOA-based sinusoidal oscillators retaining independent control of oscillation frequency even under the influence of parasitic impedances. Analog Integr. Circuits Process..

[31] Senani R., Singh V.K. (1996). Novel single-resistance-controlled-oscillator configuration using current-feedback-amplifiers. IEEE Trans. Circuits Syst. I Fundam. Theory Appl..

[32] Bhaskar D.R., Senani R. (2006). New CFOA-based single-element-controlled sinusoidal oscillators. IEEE Trans. Instrum. Meas..

[33] Andreani P., Wang X. (2004). On the phase-noise and phase-error performances of multiphase LC CMOS VCOs. IEEE J. Solid-State Circuit.

[34] Razavi B. (1996). A study of phase noise in CMOS oscillators. IEEE J. Solid State Circuits.

